# Proposed Modification of Staging for Distal Cholangiocarcinoma Based on the Lymph Node Ratio Using Korean Multicenter Database

**DOI:** 10.3390/cancers12030762

**Published:** 2020-03-24

**Authors:** Yunghun You, Yong Chan Shin, Dong Wook Choi, Jin Seok Heo, Sang Hyun Shin, Naru Kim, Kee-Taek Jang, Hongbeom Kim, Chang-Sup Lim, Sun Hee Chang, Kang Min Han, In Woong Han

**Affiliations:** 1 Department of Surgery, Samsung Medical Center, Sungkyunkwan University School of Medicine, Seoul 06351, Korea; syker80@gmail.com (Y.Y.); dw7722.choi@samsung.com (D.W.C.); jinseok.heo@samsung.com (J.S.H.); surgeonssh@gmail.com (S.H.S.); 2 Department of Surgery, Ilsan Paik Hospital, Inje University College of Medicine, Goyang 10380, Korea; eggnut81@gmail.com; 3 Department of Surgery, Asan Medical Center, University of Ulsan College of Medicine, Seoul 05505, Korea; naroo0107@naver.com; 4 Department of Pathology and Translational Genomics, Samsung Medical Center, Sungkyunkwan University School of Medicine, Seoul 06351, Korea; kt12.jang@samsung.com; 5 Department of Surgery, Seoul National University College of Medicine, Seoul 03080, Korea; surgeonkhb@gmail.com; 6 Department of Surgery, Seoul Metropolitan Government - Seoul National University Boramae Medical Center, Seoul National University College of Medicine, Seoul 07061, Korea; limcs7@gmail.com; 7 Department of Pathology, Ilsan Paik Hospital, Inje University College of Medicine, Goyang 10380, Korea; changsh@paik.ac.kr; 8 Department of Pathology, Dongguk University Ilsan Hospital, Goyang 10326, Korea; kiekie53@hanmail.net

**Keywords:** American Joint Committee on Cancer 8th edition, distal cholangiocarcinoma, modification of staging, validation

## Abstract

The 8th American Joint Committee on Cancer (AJCC) staging system for distal cholangiocarcinoma (DCC) included a positive lymph node count (PLNC), but a comparison of the prognostic predictive power of PLNC and lymph node ratio (LNR) is still under debate. This study aimed to compare various staging models made by combining the abovementioned factors, identify the model with the best predictive power, and propose a modified staging system. We retrospectively reviewed 251 patients who underwent surgery for DCC at four centers. To determine the superiority of various staging models for predicting overall OSR, Akaike information criterion (AIC), Bayesian information criterion (BIC), AIC correction (AICc), and Harrell’s C-statistic were calculated. In multivariate analysis, age (*p* = 0.003), total lymph node count (*p* = 0.033), and revised T(LNR)M staging (*p* < 0.001) were identified as independent factors for overall survival rate. The predictive performance of revised T (LNR) M staging (AIC: 1288.925, BIC: 1303.377, AICc: 1291.52, and Harrell’s C statics: 0.667) was superior to other staging system. A modified staging system consisting of revised T category and LNR predicted better overall survival of DCC than AJCC 7th and AJCC 8th editions. In the future, external validation of the proposed new system using a larger cohort will be required.

## 1. Introduction

Cholangiocarcinoma (CCC) is a malignant disease that occurs along the biliary tract and is known to constitute 3% of all gastrointestinal cancers [1]. Distal cholangiocarcinoma (DCC) refers to CCC that occurs from the point where the cystic duct joins the common hepatic duct to the ampulla of Vater [2]. Previous studies have reported poor prognosis after surgical treatment of DCC [3,4]. The American Joint Committee on Cancer (AJCC) TNM classification is widely used as a staging system to predict this dismal prognosis of the disease. Recently, the 8th edition of the AJCC [5] has undergone several changes compared to the previous (7th) edition. First, the obscure anatomical landmark called bile duct wall invasion, which was the boundary separating T2 and T1, was discarded. Instead, the T category was categorized, using objective figures expressed as the depth of invasion (DOI) [6], which was defined as the distance from the basal lamina of the adjacent normal epithelium to the deepest infiltrating tumor cells. Applying this definition, the T category is subdivided as follows: T1 (DOI < 5 mm), T2 (DOI 5–12 mm), T3 (DOI > 12 mm), and T4 (tumor involves the celiac axis or the superior mesenteric artery). Second, the group with positive regional lymph nodes that were N1 in AJCC 7th edition was subdivided into two groups, according to the positive lymph node count (PLNC) in the 8th edition. As a result, metastases in 1–3 regional lymph nodes were classified as N1, and patients with four or more metastases as N2. Validation of this new AJCC 8th staging system was performed through a single institution retrospective study [7]. It was shown that the 8th edition can predict a patient’s prognosis more accurately than the 7th edition.

At this point, however, there are some points to reconsider for the current staging system. First, a multicenter study of nearly 300 cohorts suggested cutoff values (≤ 3 mm, 3–10 mm, > 10 mm) for DOI different from the current system [8]. This cohort number is more than studies supporting the current cutoff value of the T stage [9,10]. As to N category, recent studies have reported that lymph node ratio (LNR), which is defined as the ratio of PLNC to total lymph node count (TLNC), is relatively superior to PLNC as an indicator for predicting prognosis of DCC [11,12,13]. Considering the debates about the adequacy of the T category and N category that make up the current staging system, we attempted to validate the prognostic predictive power of the AJCC 7th and 8th TNM classification in this study. In addition, the optimal cutoff value for overall survival rate (OSR) of T category, TLNC, PLNC, and LNR was also identified. Ultimately, we constructed a new TNM staging model consisting of each of the factors mentioned above and compared the prognostic predictive powers of the existing models to identify the best staging system.

## 2. Results

### 2.1. Patient Characteristics

The clinicopathologic data of 235 patients are summarized in Table 1. The median age of the patients was 65 (31–88) years, and the number of male patients was relatively higher than that of females (157 vs. 78). Preoperative bile drainage was performed in 219 (93.2%), and 232 (98.7%) patients received pancreaticoduodenectomy (PD). Lymphovascular invasion (LVI) was observed in 66.7% and perineural invasion (PNI) in 77.3%. Seventy (29.8%) patients had a poorly differentiated tumor, and the median tumor size was 2 (0.8–8.5) cm. Margin negative resection was achieved in 93.6% of patients, and cancer recurrence occurred in 133 (56.6%) patients. The median value of the tumor invasion depth was 6.0 (0.2–25.0) mm, and T3 was observed in 28 patients (11.9%) in the AJCC 8th T category, stratified according to the tumor depth. When classified by the AJCC 7th edition, 159 patients (67.7%) were classified as T3. The median TLNC and PLNC were 18 (1–64) and 2 (1–17), and the median LNR values obtained by using these two values were 0.11 (0.02–1.00). In the 7th AJCC classification, 78 patients were classified as N1. When the 8th AJCC classification was applied to them, 62 patients belonged to the N1 group, and 16 patients belonged to the N2 group. The median time of follow-up was 34.5 months (1.0–196.0), and five years cumulative OSR was 49.4%.

### 2.2. Optimal Cutoff Value for the T Category

**Figure 1:**
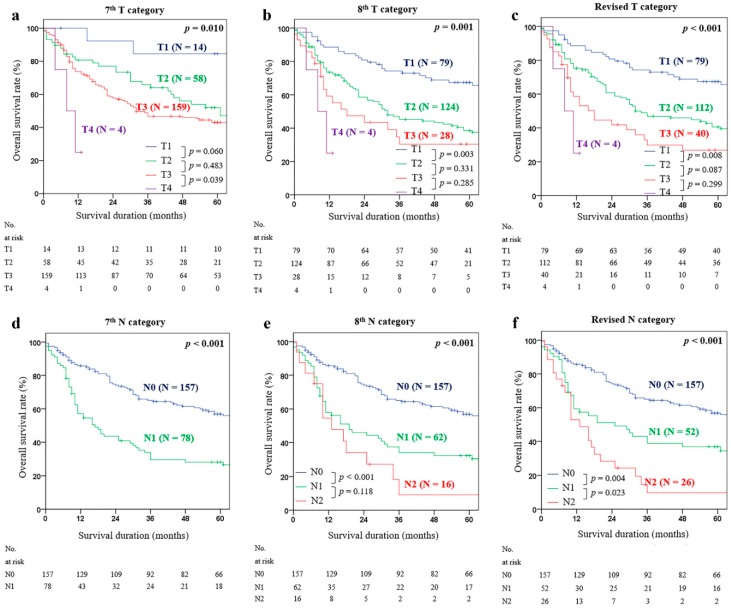
Kaplan–Meier survival analysis of five-year cumulative OSR (overall survival rate), according to (**a**) T category of the AJCC 7th classification, (**b**) T category of the AJCC 8th classification, (**c**) revised T category, (**d**) N category of the AJCC 7th classification, (**e**) N category of the AJCC 8th classification, and (**f**) revised N category.

We compared the χ^2^ score by moving from 10 to 15 mm, to obtain the cutoff value of the tumor invasion depth that best distinguishes T2 and T3. As a result, when T2 and T3 were separated based on 10 mm (T category-a), the χ^2^ score was the highest (Table 2). The new T category using this optimal cutoff value was defined as the ‘Revised T category’, which stratified T1, T2, and T3 as follows: T1 (<5 mm), T2 (5–10 mm), and T3 (>10 mm). In the 7th and 8th AJCC classifications, the survival curves between the T2 and T3 were not significantly separated (52.0% vs. 43.0%; *p* = 0.483 and 38.5% vs. 30.4%; *p* = 0.331) (Figure 1a,b). In the Revised T category, the five-year cumulative OSR of T2 and T3 showed marginally significant differences (40.6% vs. 26.9%; *p* = 0.087) (Figure 1c).

### 2.3. Optimal Cutoff Value for the TLNC, PLNC, and LNR

A stepwise algorithm was used to obtain optimal cutoff values for TLNC, PLNC, and LNR (Table 3). In the univariate analysis, the optimal value of TLNC (optimal TLNC) was found to have the highest χ^2^ when the cutoff value was set to 13. A univariate cox proportional regression analysis was also performed on the other variables, to determine if the TLNC could affect the prognosis even when adjusted with other factors affecting OSR. In multivariate analysis with age, LVI, tumor differentiation, and revised T category, which were found to affect OSR in univariate Cox proportional regression analysis, TLNC ≥ 13 was significant as optimal cutoff (HR 0.597, 95% CI 0.411–0.866; *p* = 0.007).

PLNC-b (0 vs. 1–2 vs. ≥ 3) showed the highest χ^2^ in univariate analysis (χ^2^ = 33.963; *p* < 0.001) (Table 3). In the multivariate analysis with age, LVI, tumor differentiation, and revised T category, PLNC-b (0 vs. 1–2 vs. ≥ 3) was also significant (*p* < 0.001). The new N category using this optimal cutoff value for PLNC was defined as the ‘Revised N category’. For the AJCC 8th classification, there was no significant difference between the five-year cumulative OSR of N1 and N2 (32.3% vs. 9.1%; *p* = 0.118) (Figure 1e). When we applied the revised N category, the five-year cumulative OSR between N1 and N2 showed a significant difference (36.9% vs. 9.7%; *p* = 0.023) (Figure 1f).

For LNR, χ^2^ of LNR-c (0 vs. > 0 to < 0.1 vs. ≥ 0.1) was found to be the highest in the univariate analysis (χ^2^ = 40.812; *p* < 0.001) (Table 3). In the multivariate analysis with age, LVI, tumor differentiation, and revised T category, LNR-c was also statistically significant (*p* < 0.001).

### 2.4. Multivariable Analysis for OSR

We constructed a multivariate model that includes the factors described below, to identify the independent factors affecting OSR: age, sex, preoperative bile drainage, operation type, operating time, tumor size, LVI, PNI, tumor differentiation, margin status, AJCC 7th T category, AJCC 7th N category, AJCC 8th T category, AJCC 8th N category, revised T category, revised N category, optimal TLNC, LNR-c, AJCC 7th TNM staging, AJCC 8th TNM staging, revised TNM staging, and revised T(LNR-c)M staging. Multivariate analysis using this model showed that age (HR 1.692, 95% CI 1.195–2.396; *p* = 0.003), optimal TLNC (HR 0.668, 95% CI 0.461–0.969; *p* = 0.033), and revised T (LNR-c) staging (stage I as the reference: HR for stage IIA 1.559, 95% CI 0.968–2.511; *p* = 0.068, HR for stage IIB 1.723, 95% CI 1.000–2.969; *p* = 0.050, HR for IIIA 4.606, 95% CI 2.835–7.481; *p* < 0.001 and HR for stage IIIB 8.575, 95% CI 2.535–29.002; *p* = 0.001) were independent prognostic factors (Table 4).

### 2.5. Comparison of Predictive Power of Each Staging Model (AJCC 7th TNM Staging, AJCC 8th TNM Staging, Revised TNM Staging, and Revised T(LNR-c)M Staging)

AIC, BIC, AICc, and C-statistics values for the AJCC 7th TNM staging, AJCC 8th TNM staging, revised TNM staging, and revised T (LNR-c) M staging were summarized in Table 5. Among these, the revised T(LNR-c)M-staging model had the lowest AIC (1288.925), BIC (1303.377), and AICc (1291.592) values. In contrast, the model showed the highest C-statistic (0.667).

## 3. Discussion

To determine the best treatment modality, an accurate assessment of the patient’s prognosis after surgery is essential. TNM staging is the most widely accepted and clinically used system in determining the prognosis of cancers occurring in almost all gastrointestinal tracts. Recently, the eighth edition of the AJCC TNM classification was published, and a retrospective single center study was conducted to validate the changed staging system for DCC [7]. In the study, the application of T category defined by DOI and N category with three-tier classification showed better separation of survival curves of each prognostic group than the conventional system. The concept of DOI stems from consideration of the unique anatomical structure and arrangement of components of the extrahepatic bile duct, which is different from other GI tracts [14]. Indeed, several studies have shown that this concept is clinically superior to existing T-stage criteria based on the obscure anatomical layer of extrahepatic bile duct [8,9,10]. The cutoff value that distinguishes T2 and T3 in this study was 10 mm, which is different from 12 mm in the 8th edition of AJCC. Nevertheless, this study reinforces the evidence that the concept of DOI can provide better prognostic predictive power in the T stage.

On the other hand, the debate over which of the two factors, PLNC or LNR, can predict the prognosis of DCC more accurately is still ongoing. PLNC simply refers to the number of locally metastatic lymph nodes, whereas LNR is PLNC divided by TLNC. Theoretically, the PLNC itself is more exposed to bias than LNR in that it can be influenced by the skill of the surgeon. In contrast, the findings supporting that LNR is not affected by TLNC have been reported through studies on other GI cancers [15,16,17]. For DCC, there are three single center retrospective studies that have shown that LNR actually affects prognosis independently. Kawai et al. demonstrated LNR > 0.2 affects poor prognosis of extrahepatic CCC after surgery. In the study, the enrolled cohort comprised 62 extrahepatic CCC, except for hilar CCC [18]. A cutoff value of LNR > 0.2 was also reported in another study by Oshiro et al. as an independent factor predicting poor prognosis in 60 patients with extrahepatic CCC [11]. Zhang et al. identified a four-tier classification of LNR (0 vs. 0–0.2 vs. 0.2–0.5 vs. > 0.5), which further refines the group with LNR > 0.2, as a significant risk factor in multivariate analysis [13]. In the current study, however, the three-tier classification of LNR (0 vs. > 0 to < 0.1 vs. ≥ 0.1), which was based on cutoff value = 0.1, was found to best divide the survival curve. In fact, the cutoff values of the three studies mentioned above omit the statistical effort to find the highest χ^2^ score by using the stepwise algorithm, as in current study. In addition, their LNR > 0.2 values used the same values previously suggested in studies of pancreatic cancer and ampulla of Vater cancer [19,20]. Given these points, it is questionable whether LNR > 0.2 is an appropriate cutoff value for LNR in predicting the prognosis of DCC. Further research will be needed to find the appropriate cutoff value of LNR, using refined large-scale cohorts and careful statistical analysis.

One noteworthy aspect of this study is that TLNC is found to be an independent factor in predicting survival for DCC. There has been one study showing that TLNC affects DCC survival in univariate analysis [18], but there have been no reports of significant impacts on multivariate analysis. In this study, the optimal cutoff value was TLNC ≥ 13, which is a comparable result compared to TLNC ≥ 10–11 suggested in other studies [18,21,22]. Higher TLNCs can escape the risk of under-staging by increasing the accuracy of staging for N categories. It can also be expected to ensure a better prognosis by reducing the likelihood of metastatic lymph nodes that may remain. Despite the criticism that TLNC may be influenced by the surgical policy of each institution and specimen handling by pathologists, achieving adequate TLNC is expected to enable accurate cancer staging and provide clinical benefits for the patient’s postoperative outcomes.

The staging model proposed in this study is meaningful in that it introduces LNR instead of the existing N category. As far as we know, this is the first study to compare the predictive power of the AJCC TNM staging system for DCC and the new staging system including LNR. We assumed that the LNR model predicted the prognosis better than the existing staging model composed of PLNC. Using sophisticated statistical methods, we actually demonstrated that revised T (LNR-c) M staging using LNR can predict the prognosis of DCC more accurately than other staging models. The value of 0.667, Harrell’s C-statistics of the newly revised T (LNR-c) M-staging model, was acceptable.

This study has several limitations. First, there is a flaw in the research design itself, which is analyzed by retrospective data collected from multicenters for about 10 years. Changes in the operator’s proficiency over time could not rule out a bias in the study. In addition, although the specimens were reviewed to measure DOI of tumor according to the AJCC 8th edition, there may have been some differences in specimen handling in each hospital. Second, despite the multicenter research design, there is a limitation that almost all of the data are from a single tertiary institution. Thus, validation of the new staging system must be performed in the future, using large data from other tertiary institutions.

## 4. Materials and Methods

### 4.1. Patients and Data Collection

Using the multicenter database, we identified 251 patients who underwent surgery for DCC during 2002–2012. Four Korean hospitals provided data on DCC based on medical records. Data from the following institute was retrospectively analyzed: Samsung Medical Center (*n* = 172), Seoul National University Boramae Hospital (*n* = 40), Inje University Ilsan Paik Hospital (*n* = 26), and Dongguk University Ilsan Hospital (*n* = 13). Of these patients, we excluded 16 (6.4%) patients, including 10 with no follow up data, 4 with stage of Tis, 1 with distant metastasis, and 1 with no records of total lymph node count (TLNC). The remaining 235 patients were enrolled for analysis in this study.

The analyzed variables were age, sex, preoperative bile drainage, operation type, operating time, tumor size, lymphovascular invasion (LVI), perineural invasion (PNI), tumor differentiation, margin status, tumor invasion depth, TLNC, positive lymph node count (PLNC), lymph node ratio (LNR), and tumor stage by the AJCC 7th [23]. We performed restaging to validate the 8th AJCC classification. For this purpose, the resected specimens were reviewed, and DOI was numerically analyzed. In the case of the lymph node, it was restaged by using PLNC recorded in the pathologic report. Regional lymph nodes were defined based on the Japanese system [24]. Accordingly, the range of lymph node resections performed on the same criteria in four centers was as follows: in the hepatoduodenal ligament (no. 12); anterior/posterior aspect of the pancreas and duodenum (no. 13 and 17); around the stomach (no. 1–6); around the common hepatic artery (no. 8); and along the superior mesenteric artery (station 14). PLNC was defined as the number of lymph nodes with metastases in the obtained lymph nodes, and TLNC was defined as the number of total lymph nodes found by pathologic examination in specimens. LNR was defined as PLNC divided by TLNC. We defined the overall survival time as the period from the first diagnosis to death or follow-up loss. All subjects gave their informed consent for inclusion before they participated in the study. The study was conducted in accordance with the Declaration of Helsinki, and the protocol was approved by the Ethics Committee of Institutional Review Board of Samsung Medical Center, Seoul, Republic of Korea (2020-01-126).

### 4.2. Statistical Analysis

The Kaplan–Meier method and log rank test were used to compare the overall survival rate (OSR) for each category of the AJCC 7th and AJCC 8th classifications. A stepwise algorithm is commonly used to obtain optimal cutoff values for T category, PLNC, TLNC, and LNR. In detail, the optimal cut off value for the T category was determined by the highest χ^2^ score in the univariate Cox proportional regression methods. In the case of TLNC, PLNC, and LNR, the optimal cutoff value was considered to be statistically significant in multivariate analysis with the highest χ^2^ score in the univariate cox progression model. The backward selection method of Cox proportional regression analysis was performed to identify independent factors affecting OSR.

We constructed new staging models by using this optimal cutoff value of T category, PLNC, and LNR. The predictive powers of these models and the existing staging modes (AJCC 7th and AJCC 8th editions) were compared, using the following statistical methods: Akaike information criterion [AIC = −2 log maximum likelihood + 2× degrees of freedom (df)] [25], Bayesian information criterion (BIC = −2 log maximum likelihood + log (sample size) × df) [26], corrected Akaike information criterion (AICc = −2 log maximum likelihood + [2 × n × (df + 1)/(n − df − 2)]) [27], and Harrell’s C-statistic [28]. In this study, higher values of AIC, BIC, and AICc and lower values of C-statistics were interpreted as better prediction models. We considered values to be statistically significant when the *p*-value was less than 0.05. The PASW Statistics version 23.0 (SPSS, IBM corp., Armonk, NY, USA) was used for all the abovementioned statistical analyses.

## 5. Conclusions

Modified staging system consisting of revised T category (T1: < 5 mm, T2: 5–10 mm, and T3: > 10 mm) and LNR ≥ 0.1 predicted better overall survival of DCC than the AJCC 7th and AJCC 8th editions. In the future, external validation of the proposed new system, using a larger cohort, will be required.

## Figures and Tables

**Table 1:** Clinical and pathological data of patients.

Variable	No. of Valid Records	Patients (*N* = 235)	
		No.	%
Age (years)	235		
Median		65	
Range		31–88	
Gender	235		
Male		157	66.8
Female		78	33.2
Preoperative bile drainage (n, %)	235	219	93.2
Type of operation	235		
Pancreaticoduodenectomy		232	98.7
Bile duct resection		3	1.3
Operating time (min)	235		
Median		331	
Range		195–840	
Tumor size (cm)	234		
Median		2.5	
Range		0.8–8.5	
LVI	135	90	66.7
PNI	176	136	77.3
Tumor differentiation	235		
Well/moderate		165	70.2
Poorly		70	29.8
Resection margin status	235		
R0		220	93.6
R1		12	5.1
R2		3	1.3
Recurrence	235	133	56.6
Tumor invasion depth (mm)	230		
Median		6.0	
Range		0.2–25.0	
TLNC	235		
Median		18	
Range		1–64	
PLNC	235		
Median		2	
Range		1–17	
LNR	235		
Median		0.11	
Range		0.02–1.00	
AJCC 7th T category	235		
T1		14	5.9
T2		58	24.7
T3		159	67.7
T4		4	1.7
AJCC 8th T category	235		
T1		79	33.6
T2		124	52.8
T3		28	11.9
T4		4	1.7
AJCC 7th N category	235		
N0		157	66.8
N1		78	33.2
AJCC 8th N category	235		
N0		157	66.8
N1		62	26.4
N2		16	6.8
AJCC 7th TNM staging	235		
IA		13	5.5
IB		45	19.1
IIA		96	40.9
IIB		77	32.8
III		4	1.7
AJCC 8th TNM staging	235		
I		74	31.5
IIA		77	32.8
IIB		64	27.2
IIIA		16	6.8
IIIB		4	1.7

LVI: lymphovascular invasion; PNI: perineural invasion; TLNC: total lymph node count; PLNC: positive lymph node count; LNR: lymph node ratio; AJCC: American Joint Committee on Cancer.

**Table 2:** Comparison for cutoff values of each T category.

Tumor Invasion Depth (mm)	Number of Cases (%)	Median Survival (months)	*p*-Value	χ^2^ Score
T category-a			< 0.001	18.125
T1 (<5)	79 (33.6)	131.0		
T2 (5–10)	112 (47.7)	32.0		
T3 (>10)	40 (17.0)	18.0		
T4	4 (1.7)	8.0		
T category-b			0.001	17.441
T1 (<5)	79 (33.6)	131.0		
T2 (5–11)	114 (48.5)	32.0		
T3 (>11)	38 (16.2)	16.0		
T4	4 (1.7)	8.0		
T category-c			0.001	16.059
T1 (<5)	79 (33.6)	131.0		
T2 (5–12)	114 (52.8)	32.0		
T3 (>12)	38 (11.9)	18.0		
T4	4 (1.7)	8.0		
T category-d			0.001	15.844
T1 (<5)	79 (33.6)	131.0		
T2 (5–13)	114 (53.2)	32.0		
T3 (>13)	38 (11.5)	16.0		
T4	4 (1.7)	8.0		
T category-e			0.002	15.263
T1 (<5)	79 (33.6)	131.0		
T2 (5–14)	114 (54.5)	31.0		
T3 (>14)	38 (10.2)	18.0		
T4	4 (1.7)	8.0		
T category-f			0.001	15.447
T1 (<5)	79 (33.6)	131.0		
T2 (5–15)	114 (59.6)	30.0		
T3 (>15)	38 (5.1)	-		
T4	4 (1.7)	8.0		

**Table 3:** Comparison for cutoff values of each total lymph node count (TLNC), positive lymph node count (PLNC), and lymph node ratio (LNR).

	Number of Cases (%)	Univariate Analysis			Multivariate Analysis		
		HR	*p*-Value	χ^2^ Score	HR	95% CI	*p*-Value
TLNC							
≥2	233 (99.1)	0.249	0.171	2.194	0.266	0.035–2.014	0.200
≥3	227 (96.6)	1.032	0.951	0.004	0.809	0.295–2.223	0.682
≥4	222 (94.5)	0.703	0.336	0.935	0.325	0.336–1.436	0.325
≥5	219 (93.2)	0.729	0.337	0.928	0.660	0.343–1.271	0.214
≥6	214 (91.1)	0.726	0.274	1.205	0.671	0.447–0.897	0.177
≥7	205 (87.2)	0.683	0.117	2.481	0.598	0.369–0.971	0.038
≥8	204 (86.8)	0.663	0.084	3.018	0.584	0.363–0.940	0.027
≥9	199 (84.7)	0.693	0.105	2.657	0.639	0.408–1.000	0.050
≥10	192 (81.7)	0.653	0.043	4.159	0.608	0.400–0.923	0.020
≥11	183 (77.9)	0.630	0.020	5.484	0.594	0.400–0.884	0.010
≥12	175 (74.5)	0.648	0.024	5.164	0.601	0.409–0.884	0.010
≥ 13	167 (71.1)	0.644	0.018	5.704	0.597	0.411–0.866	0.007
≥14	156 (66.4)	0.687	0.037	4.389	0.649	0.454–0.927	0.017
≥15	148 (63.0)	0.714	0.057	3.669	0.656	0.462–0.933	0.019
PLNC-a			<0.001	23.747			0.007
0	157 (66.8)	1			1		
1	34 (14.5)	2.017	0.003		1.680	1.025–2.752	0.040
≥2	44 (18.7)	2.501	<0.001		2.070	1.284–3.337	0.003
PLNC-b			<0.001	33.963			<0.001
0	157 (66.8)	1			1		
1–2	52 (22.1)	1.820	0.004		1.536	0.980–2.408	0.062
≥3	26 (11.1)	3.654	<0.001		2.968	1.742–5.057	<0.001
PLNC-c			<0.001	28.769			<0.001
0	157 (66.8)	1			1		
1–3	62 (26.4)	2.030	<0.001		1.667	1.089–2.550	0.019
≥4	16 (6.8)	3.654	<0.001		3.144	1.689–5.854	<0.001
PLNC-d			<0.001	24.628			0.005
0	157 (66.8)	1			1		
1–4	66 (28.1)	2.148	<0.001		1.749	1.157–2.657	0.008
≥5	12 (5.1)	3.138	0.001		2.657	1.315–5.371	0.006
PLNC-e			<0.001	23.470			0.008
0	157 (66.8)	1			1		
1–5	70 (29.8)	2.203	<0.001		1.793	1.189–2.706	0.005
≥6	8 (3.4)	2.909	0.007		2.425	1.082–5.434	0.031
PLNC-f			<0.001	23.648			0.004
0	157 (66.8)	1			1		
1–6	74 (31.5)	2.219	<0.001		1.793	1.195–2.691	0.005
≥7	4 (1.7)	3.275	0.021		3.439	1.206–9.811	0.021
PLNC-g			<0.001	27.934			0.002
0	157 (66.8)	1			1		
1–7	75 (31.9)	2.199	<0.001		1.749	1.210–2.711	0.004
≥8	3 (1.3)	6.177	0.002		2.657	1.600–19.170	0.007
LNR-a			<0.001	30.916			<0.001
0	157 (66.8)	1			1		
>0 to 0.05	9 (3.8)	0.870	0.786		0.925	0.338–2.534	0.879
≥ 0.05	96 (29.7)	2.594	<0.001		2.530	1.772–3.612	<0.001
LNR-b			<0.001	33.373			<0.001
0	157 (66.8)	1			1		
>0 to 0.07	22 (9.4)	1.288	0.416		0.969	0.508–1.847	0.924
≥0.07	56 (23.8)	2.862	<0.001		2.638	1.734–4.014	<0.001
LNR-c			<0.001	40.812			<0.001
0	157 (66.8)	1			1		
>0 to 0.1	33 (14.0)	1.382	0.208		1.099	0.642–1.881	0.731
≥0.1	45 (19.2)	3.400	3.400		3.254	2.078–5.095	<0.001
LNR-d			<0.001	36.183			<0.001
0	157 (66.8)	1			1		
>0 to 0.2	59 (25.1)	1.888	0.00		1.518	1.015–2.410	0.061
≥0.2	19 (8.1)	4.209	<0.001		3.912	2.303–7.091	<0.001
LNR-e			<0.001	33.438			<0.001
0	157 (66.8)	1			1		
>0 to 0.3	69 (28.9)	2.068	<0.001		1.166	1.100–2.523	0.016
≥0.3	9 (4.3)	5.416	<0.001		5.184	2.479–10.854	<0.001
LNR-f			< 0.001	25.466			0.001
0	157 (66.8)	1			1		
>0 to 0.4	72 (30.6)	2.172	<0.001		1.756	1.165–2.645	0.007
≥0.4	6 (2.6)	3.810	0.002		3.878	1.627–9.248	0.002
LNR-g			<0.001	24.847			0.002
0	157 (66.8)	1			1		
>0 to 0.5	73 (31.1)	2.191	0.001		1.780	1.184–2.676	0.006
≥0.5	5 (2.1)	3.746	0.005		3.702	1.448–9.462	0.006
LNR-e			<0.001	25.853			0.002
0	157 (66.8)	1			1		
>0 to 0.6	74 (31.5)	2.194	<0.001		1.795	1.197–2.693	0.005
≥0.6	4 (1.7)	4.490	0.004		4.156	1.465–11.792	0.007
LNR-f			<0.001	23.606			0.004
0	157 (66.8)	1			1		
>0 to 0.7	76 (32.3)	2.238	<0.001		1.812	1.209–2.716	0.004
≥0.7	2 (0.9)	3.827	0.062		4.26	1.032–17.620	0.045

HR: hazard ratio; CI: confidence interval; TLNC: total lymph node count; PLNC: positive lymph node count; LNR: lymph node ratio.

**Table 4:** Univariate and multivariate analysis of risk factor for overall survival rate (OSR).

	Univariate Analysis			Multivariate Analysis		
	HR	95% CI	*p*-Value	HR	95% CI	*p*-Value
Age (years)			0.015			0.003
<65	1			1		
≥65	1.905	1.129–3.213		1.692	1.195–2.396	
Gender			0.425			
Male	1					
Female	1.252	0.721–2.173				
Preoperative bile drainage			0.581			
No	1					
Yes	1.330	0.482–3.676				
Operation type			0.413			
Pancreaticoduodenectomy	1					
Bile duct resection	0.379	0.034–4.236				
Operating time (min)			0.957			
<350	1					
≥350	0.986	0.582–1.669				
Tumor size (cm)			0.874			
<3	1					
≥3	0.958	0.562–1.631				
LVI			0.014			0.401
No	1					
Yes	1.973	1.143–3.405				
PNI			0.419			
No	1					
Yes	1.240	0.736–2.090				
Tumor differentiation			0.034			0.221
Well/moderate	1					
Poorly	1.877	1.046–3.369				
Resection margin status			0.416			
R0	1					
R1/R2	1.577	0.522–4.767				
Revised T category			<0.001			0.494
T1 (<5)	1					
T2 (5–10)	1.716	1.144–2.575	0.009			
T3 (>10)	2.591	1.567–4.282	<0.001			
T4	6.193	1.863–20.588	0.003			
Optimal TLNC			0.018			0.033
<13	1			1		
≥13	0.644	0.447–0.927		0.668	0.461–0.969	
Revised N category			<0.001			0.731
0	1					
1–2	1.820	1.213–2.732	0.004			
≥3	3.654	2.271–5.880	<0.001			
LNR-c			<0.001			0.553
0	1					
>0 to < 0.17	1.731	1.151–2.597	0.008			
≥0.17	4.408	2.727–7.126	<0.001			
AJCC 7th T category			0.019			0.160
T1	1					
T2	2.550	0.903–7.202	0.077			
T3	2.929	1.076–7.976	0.035			
T4	10.542	2.314–48.031	0.002			
AJCC 8th T category			0.001			0.783
T1	1					
T2	1.813	1.218–2.698	0.003			
T3	2.369	1.342–4.181	0.003			
T4	6.316	1.846–20.393	0.003			
AJCC 7th N category			<0.001			0.553
N0	1					
N1	2.270	1.605–3.210				
AJCC 8th N category			<0.001			0.670
N0	1					
N1	2.030	1.394–2.956	<0.001			
N2	3.654	2.047–6.522	<0.001			
AJCC 7th TNM staging			<0.001			0.307
IA	1					
IB	2.517	0.756–8.416	0.134			
IIA	2.816	0.876–2.816	0.082			
IIB	5.940	1.855–19.023	0.003			
III	14.035	2.777–70.966	0.001			
AJCC 8th TNM staging			<0.001			0.244
IA	1					
IIA	1.752	1.101–2.789	0.018			
IIB	2.400	1.506–3.824	<0.001			
IIIA	4.964	2.612–9.434	<0.001			
IIIB	9.830	3.371–28.662	<0.001			
Revised TNM staging			<0.001			0.806
IA	1					
IIA	1.761	1.104–2.810	0.018			
IIB	2.140	1.318–3.476	0.002			
IIIA	4.940	2.851–8.560	<0.001			
IIIB	7.382	2.201–24.765	0.001			
Revised T(LNR-c)M staging	3.810		<0.001			<0.001
IA	1			1		
IIA	1.683	1.049–2.698	0.031	1.559	0.968–2.511	0.068
IIB	2.346	1.472–3.738	<0.001	1.723	1.000–2.969	0.050
IIIA	5.707	3.153–10.333	<0.001	4.606	2.835–7.481	<0.001
IIIB	7.420	2.211–24.897	0.001	8.575	2.535–29.002	0.001

HR: hazard ratio; CI: confidence interval; LVI: lymphovascular invasion; PNI: perineural invasion; TLNC: total lymph node count; PLNC: positive lymph node count; LNR: lymph node ratio.

**Table 5:** Comparison of predictive power of each staging model.

Model	AIC	BIC	AIC_C_	Harrell’s C-Statistics
AJCC 7th staging	1298.281	1309.842	1300.753	0.562
AJCC 8th staging	1297.589	1312.041	1300.256	0.658
Revised TNM staging	1294.025	1308.477	1296.692	0.662
Revised T(LNR-c)M staging	1288.925	1303.377	1291.592	0.667

AIC: Akaike information criterion; BIC: Bayesian information criterion; AIC_C_: corrected Akaike information criterion.
